# microRNA-guided immunity against respiratory virus infection in human and mouse lung cells

**DOI:** 10.1242/bio.060172

**Published:** 2024-06-17

**Authors:** Ayaka Shibamoto, Yoshiaki Kitsu, Keiko Shibata, Yuka Kaneko, Harune Moriizumi, Tomoko Takahashi

**Affiliations:** ^1^ Department of Biochemistry and Molecular Biology, Faculty of Science, Saitama University, Saitama 338-8570, Japan; ^2^ Department of Biochemistry and Molecular Biology, Graduate School of Science and Engineering, Saitama University, Saitama 338-8570, Japan

**Keywords:** RNA silencing, RNA–RNA interaction, Lungs, MicroRNA, Respiratory virus

## Abstract

Viral infectivity depends on multiple factors. Recent studies showed that the interaction between viral RNAs and endogenous microRNAs (miRNAs) regulates viral infectivity; viral RNAs function as a sponge of endogenous miRNAs and result in upregulation of its original target genes, while endogenous miRNAs target viral RNAs directly and result in repression of viral gene expression. In this study, we analyzed the possible interaction between parainfluenza virus RNA and endogenous miRNAs in human and mouse lungs. We showed that the parainfluenza virus can form base pairs with human miRNAs abundantly than mouse miRNAs. Furthermore, we analyzed that the sponge effect of endogenous miRNAs on viral RNAs may induce the upregulation of transcription regulatory factors. Then, we performed RNA-sequence analysis and observed the upregulation of transcription regulatory factors in the early stages of parainfluenza virus infection. Our studies showed how the differential expression of endogenous miRNAs in lungs could contribute to respiratory virus infection and species- or tissue-specific mechanisms and common mechanisms could be conserved in humans and mice and regulated by miRNAs during viral infection.

## Teaser

**Summary:** Our studies showed how the differential expression of pulmonary miRNAs could contribute to respiratory virus infection and species- or tissue-specific mechanisms and common mechanisms conserved in humans and mice.

## INTRODUCTION

When a virus infects the human body, both innate and adaptive immune responses are initiated for protection (Alberts et al., 2022). In addition, the importance of small RNAs in defense systems has recently become apparent (Takahashi et al., 2021). Small RNAs such as small interfering RNAs (siRNAs) or microRNAs (miRNAs) bind to target RNA using sequence complementarities and degrade RNA or suppress its functions via a mechanism called RNA silencing (Bartel, 2004; Wilson and Doudna, 2013). In invertebrates and plants, RNA silencing targeting viral genomic or transcribed RNAs is essential as an antiviral defense system (Ding and Voinnet, 2007). Viral siRNAs are generated from viral double-stranded RNA (dsRNA) by an endogenous endoribonuclease, Dicer, and viral siRNAs target the complementary region of viral RNA to eliminate it from the body (Wang and Metzlaff, 2005; Leggewie and Schnettler, 2018). Thus, siRNA-guided immunity, in which viral siRNA targets viral RNA, functions in these organisms. In mammals, protein-guided immunity, such as cytokines and antibodies, and cell-guided immunity, such as natural killer (NK) cells and cytotoxic T cells, which are classified as innate or adaptive immunity, have been developed (Alberts et al., 2022). It remains unclear whether siRNA-guided antiviral immunity functions as a defense system in mammals because viral siRNAs are not detected in virus-infected somatic cells, except for infection with viruses lacking specific viral genes or infection in stem cells (Li et al., 2004, 2013, 2016; Lu and Cullen, 2004; Sullivan and Ganem, 2005; Bennasser et al., 2005; Maillard et al., 2013, 2019; Guo et al., 2019). Furthermore, whether protein- or cell-guided immunity acts as a tradeoff with siRNA-guided antiviral immunity also remains unclear (Takahashi and Ui-Tei, 2020). However, recent studies showed that the interaction between viral RNAs and endogenous miRNAs regulates viral infectivity; endogenous miRNAs not only regulate endogenous gene expression (Takahashi et al., 2021; Cullen, 2013; Girardi et al., 2018) but also can target viral RNA directly (Vaddadi et al., 2023), while viral RNAs function as a sponge of endogenous miRNAs and result in the upregulation of their original target genes (Bartoszewski et al., 2020; Pepe et al., 2022; Li et al., 2022).

MiRNAs are small non-coding RNA of approximately 22 nucleotides in length that are expressed in all human cells (Bartel, 2004; Wilson and Doudna, 2013). The human genome encodes 1917 miRNA precursors and 2656 mature miRNAs, according to the miRNA database miRBase (Kozomara et al., 2019). Mature miRNAs repress gene expression post-transcriptionally by the mechanism called RNA silencing (Bartel, 2004; Wilson and Doudna, 2013). The expression profiles of mature miRNAs differ in each cell or tissue, and a precise regulatory network is observed between miRNAs and genes in response to exogenous or endogenous stress (Ludwig et al., 2016; de Rie et al., 2017; Moore et al., 2020). miRNAs are transcribed from the genome as primary transcripts and processed sequentially into miRNA duplexes by endoribonucleases Drosha (Lee et al., 2003) and Dicer (Doi et al., 2003). The sources of viral siRNAs and endogenous miRNAs differ; however, they are incorporated into the RNA-induced silencing complex (RISC) to perform RNA silencing (Liu et al., 2004). It has been reported that aberrant expression or nucleotide substitution of specific miRNAs is associated with various diseases including infectious diseases, cancer, and neurodegenerative diseases (Li et al., 2018; Liu et al., 2021; Sonehara et al., 2022). miRNAs are expressed in all human cells, including epithelial cells, which are susceptible to viral infection. The number of miRNAs encoded in the genome tends to increase with the complexity of the organism (Table S1). Furthermore, comparative analysis of the genome and miRNA expression profile of great apes such as chimpanzees, orangutans, and gorillas have shown human-specific single nucleotide variants (SNV) of miRNAs, which were linked to human-specific gene regulation related to high-order biological functions (Gallego et al., 2016; McCreight et al., 2017).

The abundance and variety of miRNAs contribute to the probability of base pairing with viral RNAs. When a virus infects cells, viral RNAs interact with differential endogenous miRNAs in each cell. In this study, we analyzed the contribution of differential expression of miRNAs that are expressed in human or mouse lungs to infection with mouse parainfluenza virus (MPIV; also known as Sendai virus, SeV), which causes pneumonia in mice but not in humans. For comparison, we also analyzed the human parainfluenza virus (HPIV), which cause respiratory illness such as croup and pneumonia in children and the elderly. We showed that the possible interaction between viral RNAs and endogenous miRNAs are different in a species-specific manner and could function as an antiviral defense system in mammalian cells.

## RESULTS

### Endogenous miRNAs that are expressed in human or mouse lungs

Viral infectivity depends on multiple factors outside the cells, such as the receptors on the cell surface or environmental conditions (Maginnis, 2018). However, the mechanism by which endogenous miRNAs contribute to viral replication or gene expression after viral entry remains unclear. Recent studies showed that the interaction between viral RNAs and endogenous miRNAs regulates the expression of viral genes and viral replication (Vaddadi et al., 2023; Bartoszewski et al., 2020; Pepe et al., 2022; Li et al., 2022). To investigate the contribution of endogenous miRNAs after viral entry into cells, we analyzed the possible interaction between positive and negative strands of parainfluenza virus RNAs and endogenous miRNAs that are expressed in human and mouse lungs.

The mouse parainfluenza virus (MPIV) is a non-segmented, single-stranded RNA virus belonging to the family Paramyxoviridae. The genome (negative strand) encodes the N, P, M, F, HN, L, C, V, W, and Y genes, and the monocistronic viral mRNAs are transcribed (Hulo et al., 2011). MPIV attaches to sialic acids on the cell surface of host cells using the HN protein in the envelope, and the F protein fuses the membranes of the host cells and the viral envelope (Markwell and Paulson, 1980). MPIV replicates the genome in the cytoplasm, and both the genome and mRNA are localized in the cytoplasm throughout the life cycle (Takimoto and Portner, 2004). MPIV enters the body via aerosol transmission route and induces severe pneumonia in mice but not in humans (Brownstein, 1983). MPIV infection induces the production of type-I interferon, viral RNA amplification, and cell death in cultured cells. It is considered that the differences in the resistance of individual humans and mice depend on the complexity and effectiveness of the immune system. However, their regulatory factors remain unclear.

**Fig. 1.**
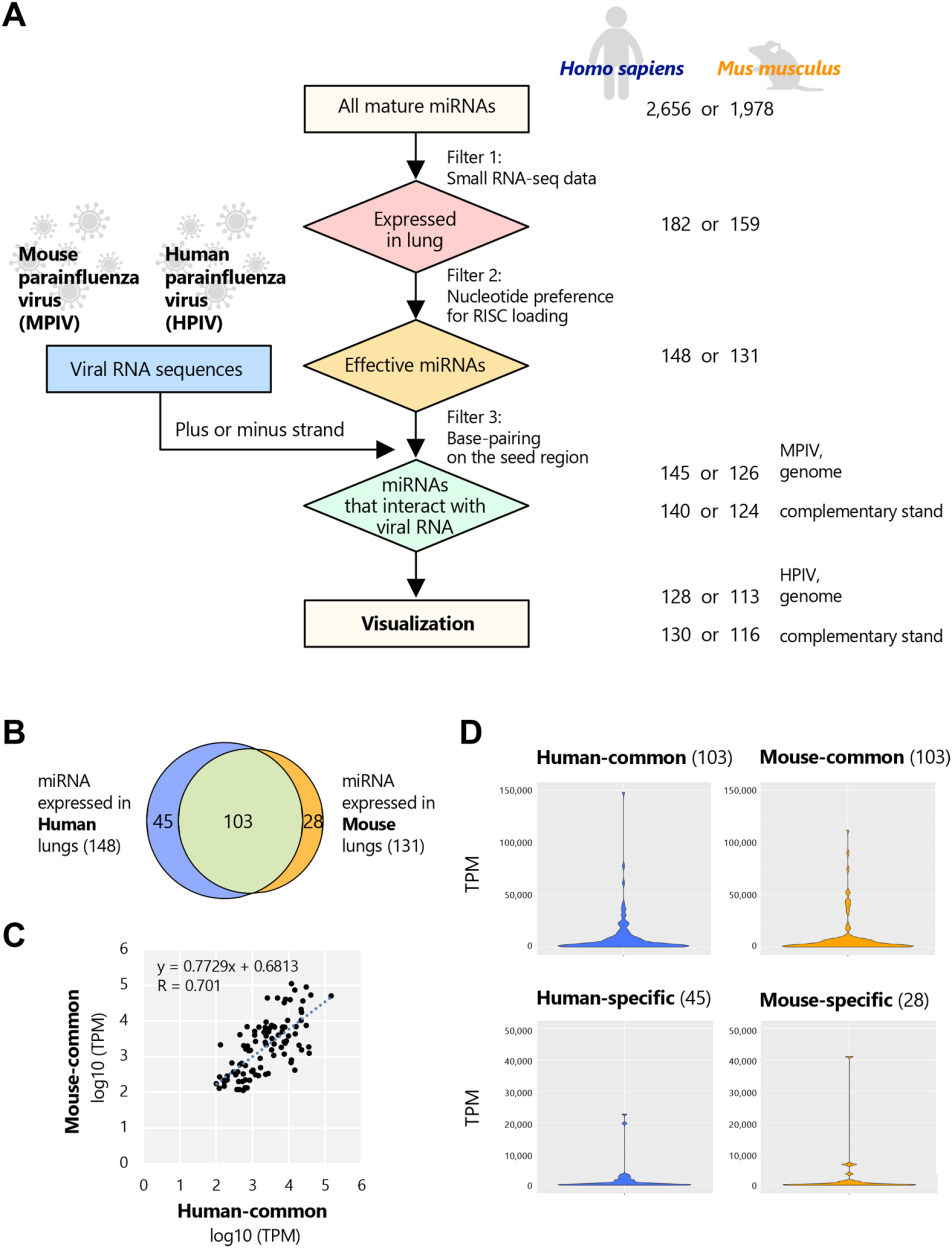
**Endogenous miRNAs that are expressed in human or mouse lungs.** (A) Small RNA-seq data were obtained from a public database, and miRNA expression profiles of human or mouse lungs were analyzed to extract miRNAs expressed in human or mouse lungs from all miRNAs registered on miRBase (Filter 1). The miRNAs that were assumed to be effective were extracted in terms of nucleotide preference at the 5′ end of the mature miRNA (Filter 2). The miRNAs that could interact with viral RNAs via the seed region in positions 2–7 from the 5′ end of the miRNA were extracted (Filter 3). Using the miRNAs that passed all filters, we performed the visualization of “viral RNA versus endogenous miRNA”. The number of human and mouse miRNAs registered on miRBase and that of miRNAs that passed each filter is shown on the right side of the flowchart. (B) The Venn diagram of the miRNAs that are expressed in the lungs and exhibited an effective sequence: 148 human miRNAs and 131 mouse miRNAs. (C) The XY plot of the expression levels (TPM) of common effective miRNAs in human or mouse pulmonary lungs. (D) The violin plots of the expression levels (TPM) of common or species-specific miRNAs in human or mouse lungs.

**Fig. 2.**
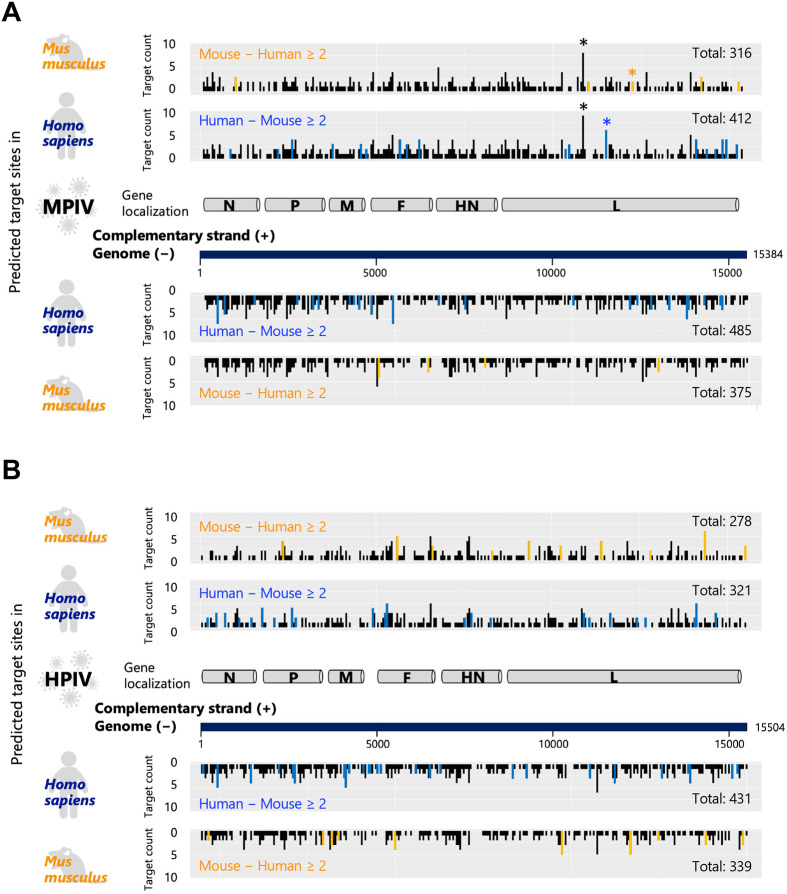
**The possible interaction between parainfluenza virus RNAs and endogenous miRNAs in human and mouse lungs.** (A) The miRNA interaction sites on the genome and its complementary strand of mouse parainfluenza virus (MPIV). (B) The miRNA interaction sites on the genome of the human parainfluenza virus (HPIV) and its complementary strand. The upper half shows the number of human or mouse miRNAs that interact with each position of the complementary strand of the viral genome (positive stand). The lower half shows the number of human or mouse miRNAs that interact with each position of the viral genome (negative strand). The positions with blue lines indicate the position with higher interaction counts in humans than in mice (human–mouse ≥2) and those with orange lines indicate the position with higher interaction counts in humans than in mice (mouse–human ≥2). The black asterisks in A show the example of positions demonstrating a similar tendency for interactions with miRNAs in humans and mice. The blue asterisk shows the example of positions with higher interaction counts in humans than in mice, and the orange asterisk shows the example of positions with higher interaction counts in mice than humans.

First, we analyzed and visualized the possible miRNA interaction sites on positive and negative strands of MPIV RNAs (Figs 1 and 2). According to the miRNA database, miRBase, 2656 and 1978 mature miRNAs are expressed in human and mouse cells, respectively (Kozomara et al., 2019). Infection with MPIV can infect both human and mouse cultured cells but induces pneumonia in mice and not in humans; therefore, we analyzed the contribution of endogenous miRNAs that are expressed in the lungs for MPIV by comparing humans and mice. Small RNA-seq data derived from human and mouse lungs were obtained from a public database and classified by age (Table S2). The miRNA expression profiles of human lungs were obtained from 15 donors without a history of disease (16–39 years old), and those of mouse lungs were obtained from 23 mice (8–16 weeks old). Several miRNAs showed donor-specific expression patterns. However, we averaged the read counts after normalization (TPM) to extract the common pattern for the young generation in both human and mouse lungs. The violin plots of TPM values for miRNAs expressed in human or mouse lungs with TPM ≥1 revealed a similar distribution between human and mouse lungs, with the majority of these miRNAs having TPM values <100 (Fig. S1). As the first filter of the analysis, we extracted the miRNAs expressed in human or mouse lungs with TPM values ≥100. The numbers of miRNAs expressed in human and mouse lungs (TPM ≥100) were 182 and 159, respectively (Fig. 1A, filter 1). As the second filter, we extracted miRNAs that were assumed to be effective in terms of nucleotide preference at the 5′ end of mature miRNAs. AGO interacts with the phosphorylated 5′ end of miRNA in the pocket of the MID domain and shows a clear bias for U or A (Frank et al., 2010). The expression level of each miRNA and the ratio of the 5′ nucleotide U or A were positively correlated (Fig. S2). The miRNA with U or A nucleotide in the 5′ position exhibits approximately 30 times higher affinity for loading onto the AGO protein compared to the miRNA with G or C nucleotide in the 5′ position (Frank et al., 2010). In addition, the violin plots of TPM values for miRNAs with G or C nucleotide in the 5′ position revealed a concave distribution centered around TPM=3000 (Fig. S3). Based on these results, a threshold of TPM ≥100 was set for miRNAs with U or A nucleotides in the 5′ position, and TPM≥3000 was set for miRNAs with G or C nucleotides in the 5′ position, presuming them to be effective miRNAs. The numbers of miRNAs that passed the second filter were 148 and 131, respectively (Fig. 1A, filter 2). Among the miRNAs that passed the second filter, the number of common miRNAs was 103, and the numbers of human- or mouse-specific miRNAs were 45 and 28, respectively (Fig. 1B). The expression levels of common effective miRNAs were correlated between human and mouse lungs (Fig. 1C, R=0.701). Violin plots of the expression levels of common or species-specific miRNAs showed a similar distribution between human and mouse lungs, indicating that the expression levels of effective pulmonary miRNAs are not different between species (Fig. 1D). As the third filter, we analyzed whether the miRNAs that passed the first two filters could form the interaction with viral RNA. miRNAs recognize the target RNA by forming base pairs via the seed region, which spans positions 2–7 from the 5′ end of the miRNA (Lewis et al., 2003). Viral nucleoproteins cover the viral genome. However, the genome and its complementary strand are generated as replication intermediates, and both strands are assumed to be accessible during replication (Trobaugh and Klimstra, 2017; Hausser et al., 2013). We searched for complementary sequences of the seed region on the genome (negative strand) and its complementary strand (positive strand) of MPIV, and HPIV for comparison. The nucleotide contents showed no significant difference between MPIV and HPIV, although HPIV may possess slightly more A/U nucleotides than MPIV (Fig. S4). Among the miRNAs that passed through the second filter, the numbers of human or mouse miRNAs that could interact with the MPIV genome were 145 and 126, respectively and the numbers with its complementary strand were 140 and 124, respectively. In contrast, those with the HPIV genome were 128 (human) and 113 (mouse) and that with its complementary strand was 130 or 116 (Fig. 1A, filter 3). Using the miRNAs that passed all three filters, we visualized the possible miRNA interaction sites with viral RNAs.

### The possible interaction between parainfluenza virus RNAs and endogenous miRNAs in human and mouse lungs

The MPIV and HPIV genomes localize and replicate in the cytoplasm (Takimoto and Portner, 2004). The MPIV or HPIV genome (negative-stranded RNA) is 15,384 or 15,504 nucleotides in length (Hulo et al., 2011), and its complementary strand (positive-stranded RNA) is transcribed as a replication intermediate or mRNA. We visualized how the genome and complementary strand of MPIV (Fig. 2A) or HPIV (Fig. 2B) could interact with endogenous miRNAs in human or mouse lungs. The vertical axis of each plot shows the total number of possible interactions with miRNA at each position. Extensive interaction with miRNAs through the genome and its complementary strand was observed in both MPIV and HPIV (Fig. 2A and B). The total counts of possible interactions with miRNAs in the MPIV genome were 485 or 375 and those in the complementary strand were 412 or 316 in human and mouse lungs, respectively. In contrast, those in the HPIV genome were 431 or 339, and those in the complementary strand were 321 and 278 in human and mouse lungs, respectively. These results suggest that both strands of MPIV or HPIV can interact with endogenous miRNAs in human lungs than in mouse lungs. Furthermore, the genome showed higher counts of possible interaction than its complementary sequences for both MPIV and HPIV.

**Fig. 3.**
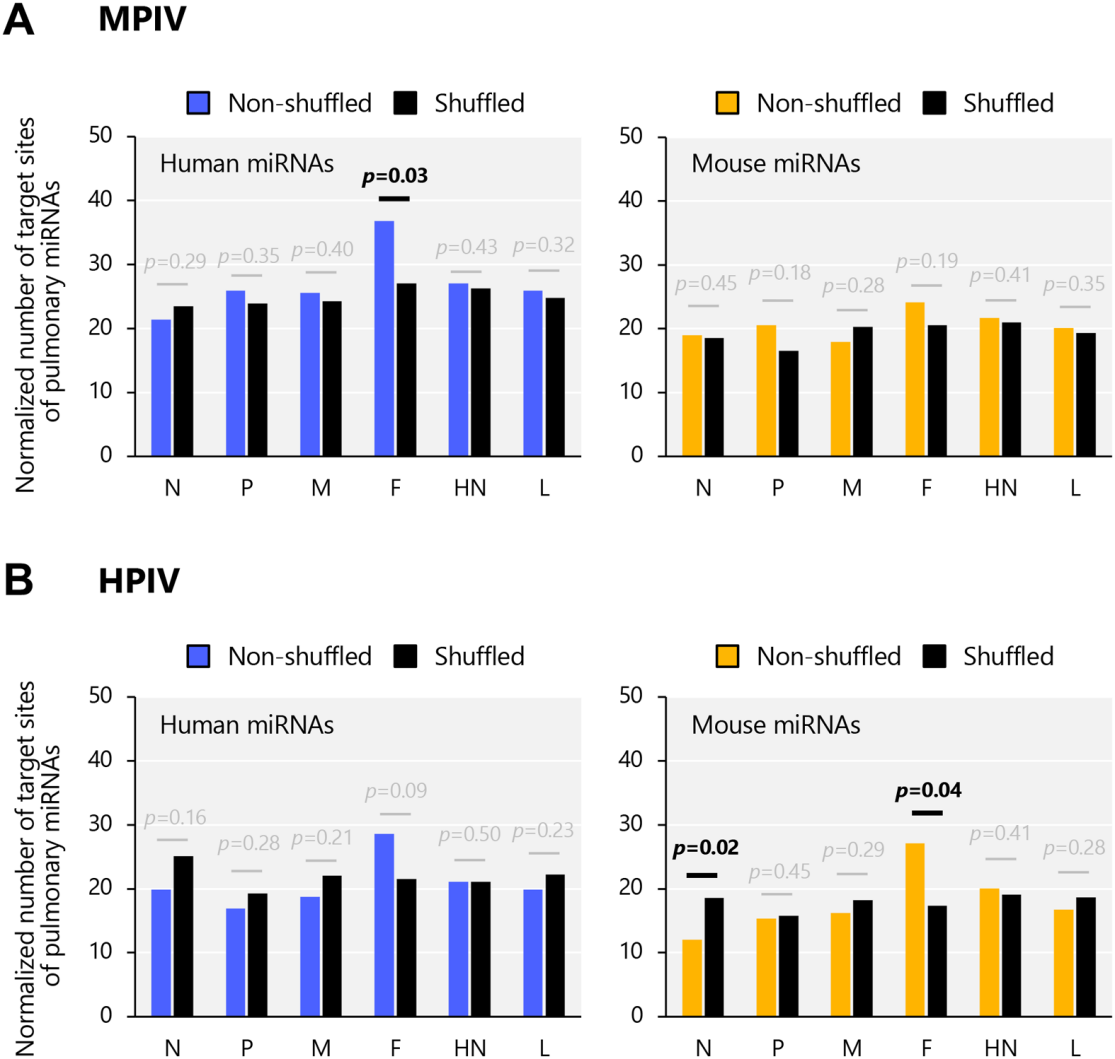
**Normalized number of target sites of pulmonary miRNAs on each transcript encoding MPIV or HPIV genes.** The sequences underwent 3-mer shuffling 10 times, and the averaged values of these shuffled sequences from 1 to 10 iterations were used as the results of the shuffled sequences. The numbers were normalized to the length of the transcripts. Statistical analysis was performed using a one-tailed, two-sample *t*-test with equal variances. (A) MPIV (B) HPIV.

Some positions showed higher counts compared with peripheral regions in both humans and mice (for example, Fig. 2A black asterisk), whereas the others showed no counts. The positions with blue lines indicate the position with higher counts in humans than in mice (human–mouse ≥2) (for example, Fig. 2A blue asterisk), indicating that these positions can interact with endogenous miRNAs in human lungs than those in mouse lungs. The orange lines indicate the position with higher counts in humans than those in mice (mouse–human ≥2) (for example, Fig. 2A orange asterisk), indicating that these positions can interact with endogenous miRNAs in mouse lungs than those in human lungs. Throughout the entire sequence, the number of positions with blue lines was higher than that with orange lines, indicating that these differences may contribute to the differential interaction between viral RNAs and endogenous miRNAs in human and mouse lungs. To determine the quantitative values of target sites for pulmonary miRNAs in each genomic region of HPIV or MPIV, we analyzed the total number of target sites on transcripts encoding N, P, M, F, HN, or L genes, normalized by the length of those transcripts. Each transcript underwent 3-mer shuffling 10 times, and the averaged values of these shuffled sequences from one to 10 iterations were used as the results of the shuffled sequence (Fig. 3). The results showed that the F gene of MPIV is not significantly targeted by mouse pulmonary miRNAs, whereas that is significantly targeted by human pulmonary miRNAs, compared with the shuffled sequence (Fig. 3A). In contrast, The F gene of HPIV is not significantly targeted by human pulmonary miRNAs, whereas that is significantly targeted by mouse pulmonary miRNAs (Fig. 3B). These results suggest the possibility that the viruses had undergone escape mutations on the F gene, which encodes a fusion protein, from being targeted by miRNAs that are expressed in the lungs of host species.

### Common or species-specific miRNAs that possess multiple interaction sites on viral RNA

**Fig. 4.**
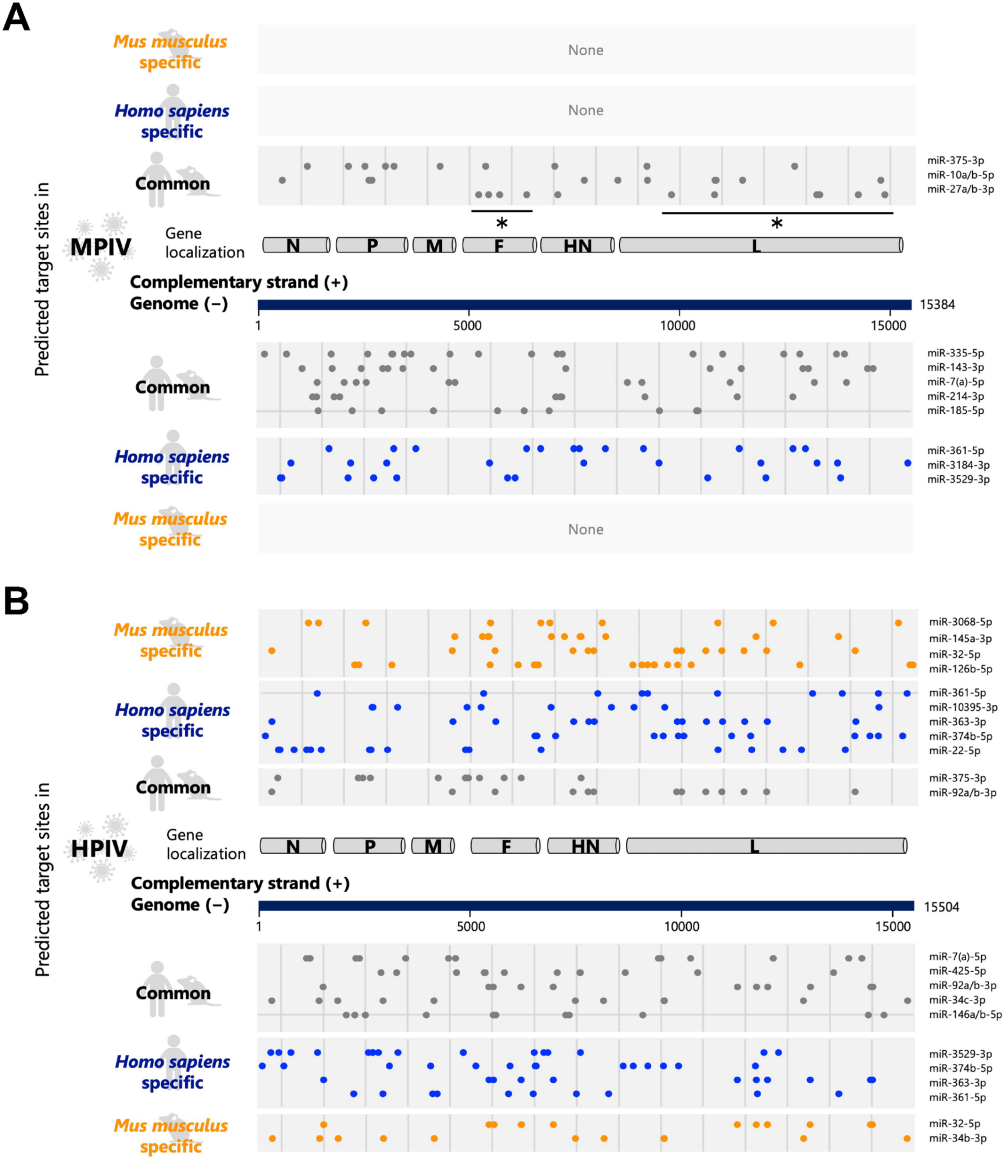
**Common or species-specific miRNAs that possess multiple interaction sites on viral RNA.** (A) The interaction sites of miRNA with multiple target sites (≥10) on the genome of MPIV (the lower half) and its complementary strand (the upper half). (B) The interaction sites of miRNA with multiple target sites (≥10) on the genome (the lower half) of HPIV and its complementary strand (the upper half). miRNAs were distinguished into three types: common miRNAs in humans and mice, human-specific miRNAs, and mouse-specific miRNAs.

Several miRNAs have multiple interaction sites in the viral genome and their complementary strands. We generated a list of miRNAs with multiple interaction sites on viral RNA (Tables S3–S6). We then visualized the interaction sites of miRNAs with multiple interaction sites (≥10) on the viral genome and its complementary strands (Fig. 4). We distinguished three types of miRNAs: miRNAs common to humans and mice, human-specific miRNAs, and mouse-specific miRNAs (Fig. 1B). Five miRNAs (miR-335-5p, −143-3p, −7(a)-5p, −214-3p, and −185-5p) and three miRNAs (miR-27a/b-3p, −10a/b-5p, and −375-3p) in humans and mice can interact with the MPIV genome and its complementary strand through 10 or more interaction sites, respectively (Fig. 4A, gray dots). In addition, The MPIV genome comprised 10 or more interaction sites of human-specific miRNAs (miR-361-5p, −3184-3p, and −3529-3p) (Fig. 4A, blue dots). Ten or more interaction sites are presumed to be sufficient as a threshold, although we do not exclude the possibility that nine or fewer interaction sites may also be functional. In contrast, the HPIV genome and its complementary strand can interact through 10 or more interaction sites with five types of miRNAs (miR-7(a)-5p, −425-5p, −92a/b-3p, −34c-3p, and −146a/b/-5p) and two types of miRNAs (miR-92a/b-3p and −375-3p) commonly found in humans and mice (Fig. 4B). In addition, The HPIV genome and its complementary strand had 10 or more interaction sites of human-specific miRNAs (miR-3529-3p, −374b-5p, −363-3p, and −361-5p in the genome; miR-22-5p, −374b-5p, −363-3p, −10395-3p, and −361-5p on the complementary strand) and mouse-specific miRNAs (miR-32-5p and −34b-3p in the genome; miR-126b-5p, −32-5p, −145a-3p, and −3068-5p on the complementary strand). Several miRNAs exhibit a position bias of interaction sites. For instance, miR-27a/b-3p does not have the interact sites in the region encoding N, P, and M genes but have in the region encoding F gene at four positions and the L gene at six positions (Fig. 4A, black asterisks), suggesting that specific miRNAs can interact viral RNA in position-specific manner.

### The sponge effect and upregulated genes induced by interaction between viral RNAs and endogenous miRNAs

**Fig. 5.**
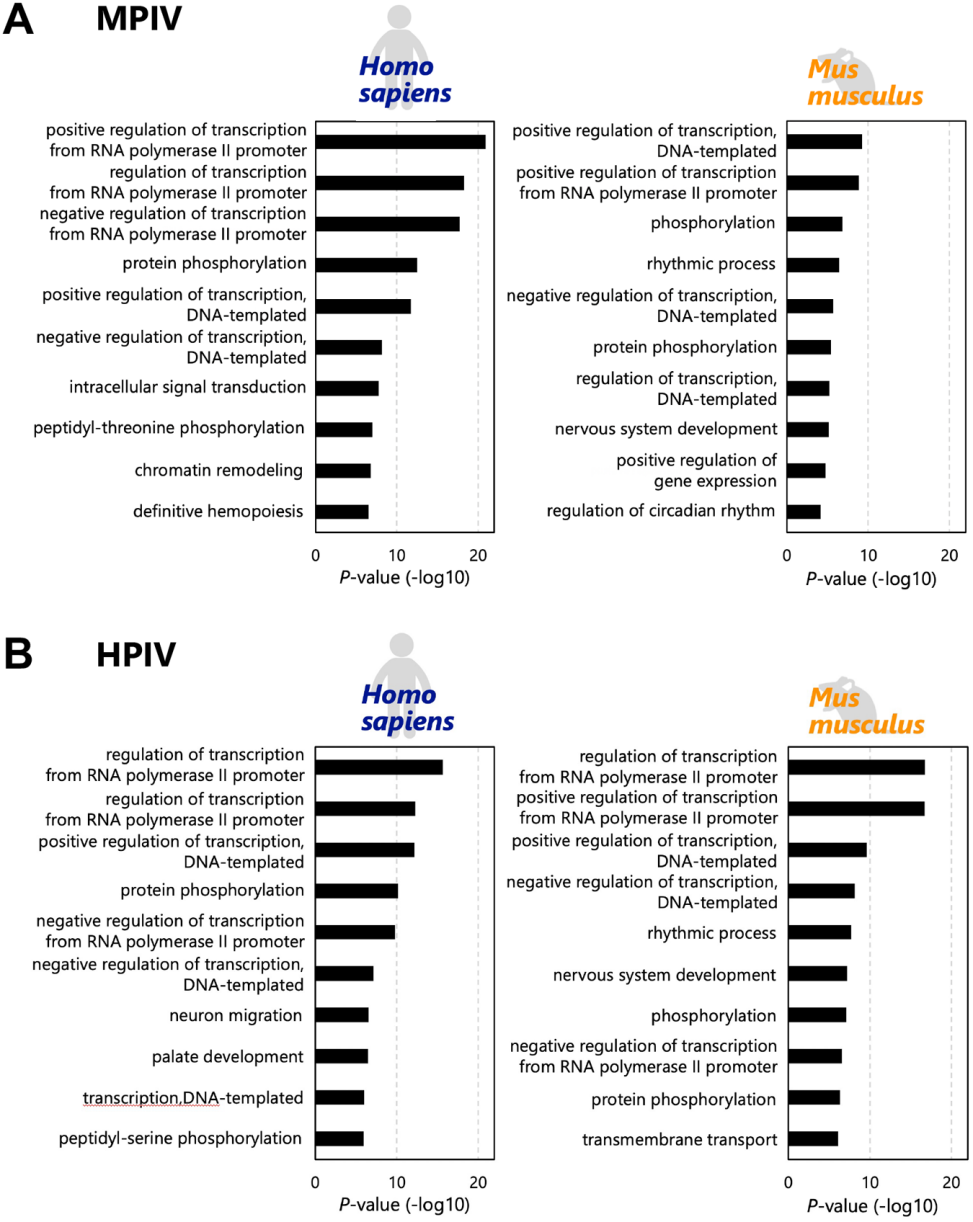
**The sponge effect and upregulated genes induced by interaction between viral RNAs and endogenous miRNAs.** (A) The gene ontology (GO) analysis of candidate genes that can be dysregulated in MPIV-infected human or mouse lungs. (B) The GO analysis of candidate genes that can be dysregulated in HPIV-infected human or mouse lungs. The genes that can be targeted by more than three miRNAs among miRNAs with multiple target sites (≥10) on the viral genome and its complementary strand were defined as the target genes that can be dysregulated.

**Fig. 6.**
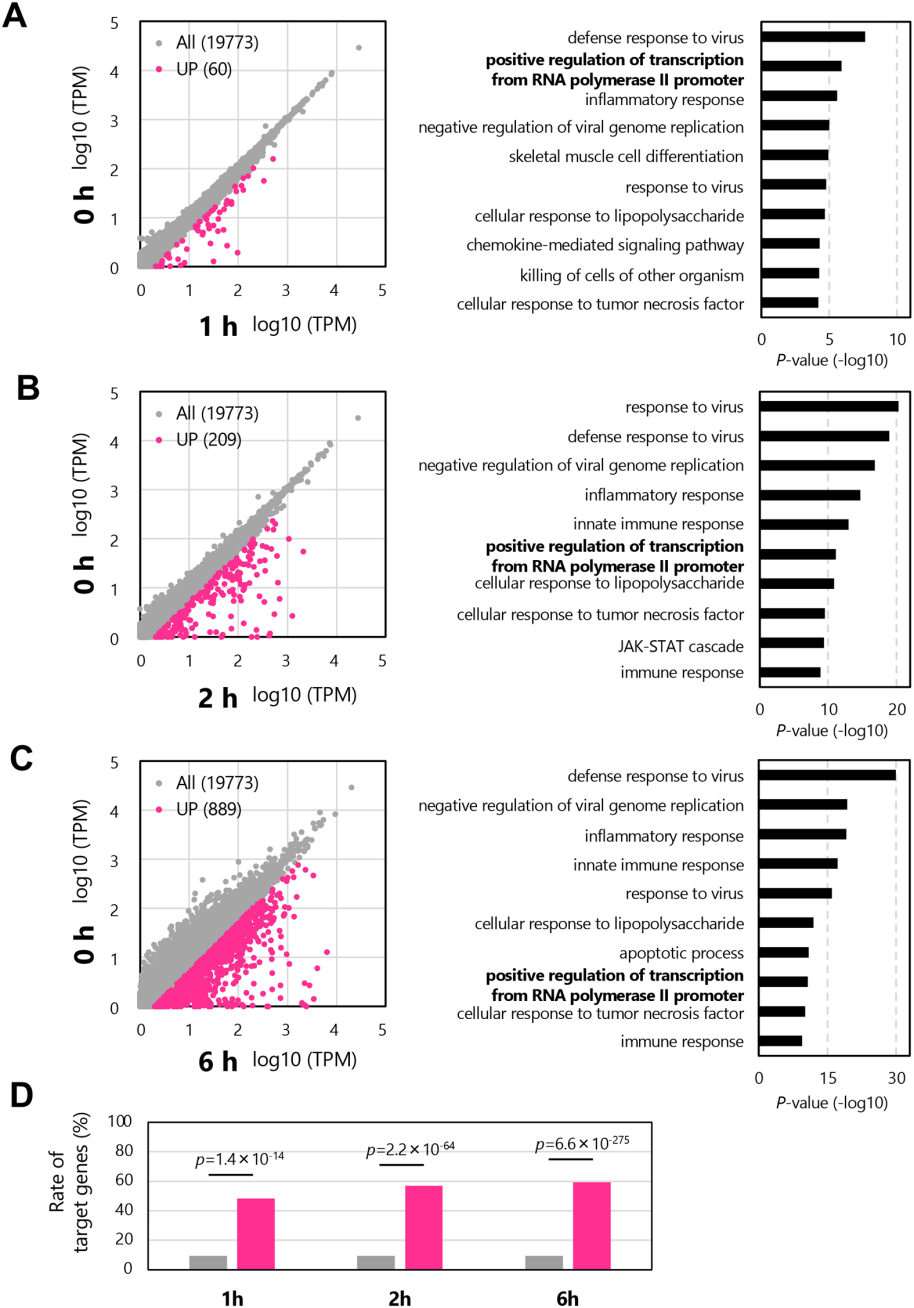
**Gene expression profiling of MPIV-infected A549 cells.** XY plot of normalized read counts (log10, TPM) of all transcripts (gray) and upregulated genes (SeV/mock >2, pink) in A549 cells collected at 1 h (A), 2 h (B), or 6 h (C) following MPIV infection and the gene ontology (GO) analysis of upregulated genes in MPIV-infected cells. (D) Rate of genes targeted by three or more pulmonary miRNAs with multiple interaction sites (≥10). Each bar represents the rate of genes targeted by three or more pulmonary miRNAs with multiple interaction sites (≥10) in all transcripts (gray bars) or in upregulated transcripts at 1, 2, or 6 h, respectively (pink bars). Statistical analysis was performed using a Fisher's test.

We analyzed how the interaction between viral RNAs and endogenous miRNAs influences endogenous gene expression in host cells. miRNAs with multiple interaction sites (Fig. 4 and Tables S3–S6) can interact with viral RNA effectively, and the viral RNAs can function as a sponge of miRNAs to induce the dysregulation (upregulation) of its original target genes (Bartoszewski et al., 2020; Pepe et al., 2022; Li et al., 2022). We performed target prediction for miRNAs with multiple interaction sites (≥10) on the viral genome and its complementary strand using TargetScan (Agarwal et al., 2015; McGeary et al., 2019) (Fig. 5). Genes targeted by three or more miRNAs with multiple interaction sites (≥10) on the viral genome and its complementary strand were defined as target genes that could be dysregulated. When MPIV infects the human lungs, 1937 candidate genes could be dysregulated. In contrast, when MPIV infects mouse lungs, 337 candidate genes could be dysregulated. Gene ontology (GO) analysis of 1937 or 337 genes showed enrichment of specific GO terms corresponding to the regulation of transcription (Fig. 5A; Tables S7 and S8). Furthermore, similar GO terms were enriched in HPIV (Fig. 5B; Tables S9 and S10). We found that the transcription regulatory factors could be upregulated by miRNA sequestration on MPIV or HPIV RNAs in both human and mouse lungs (Fig. 5). Regulation of transcription is an essential process during viral infection because the transcription of antiviral cytokines, such as type I interferon (IFN), and antiviral genes, such as IFN-stimulated genes (ISGs), must be upregulated to protect infected cells and peripheral cells (Yoneyama and Fujita, 2010). For experimental validation, we infected A549 cells which are cultured cells derived from human lungs with MPIV, and collected the cells 1 h, 2 h, or 6 h following the infection to perform RNA-seq. Using the experimentally acquired mRNA-seq data, we performed gene expression profiling of MPIV-infected A549 cells at each time point (Fig. 6 and Tables S11–S13). The results showed that 60, 209, and 889 genes were upregulated 1, 2, and 6 h following infection with MPIV (Fig. 6A–C, SeV/mock>2). Among the 60, 209, and 889 genes, 29, 119, and 528 genes were targeted by three or more miRNAs with multiple interaction sites (≥10) on the MPIV genome and its complementary strands (miR-335-5p, −143-3p, −7(a)-5p, −214-3p, −185-5p, miR-361-5p, −3184-3p, −3529-3p, miR-27a/b-3p, −10a/b-5p, and −375-3p). Among all genes, 1872 genes were targeted by three or more miRNAs with multiple interaction sites (≥10). This indicates that the overlap rates between genes upregulated by MPIV infection and the target genes of miRNAs with multiple interaction sites (≥10) on the MPIV genome and its complementary strands were 48.3%, 56.9%, and 59.4%, while that between all genes and the target genes of miRNA with multiple interaction sites (≥10) was 9.5% (Fig. 6D). The statistical analysis showed that the enrichments of target genes in upregulated transcripts during viral infection are significant. GO analysis revealed that the GO terms related to antiviral innate immune response and positive regulation of transcription were enriched at all 1 h, 2 h, and 6 h (Fig. 6A–C and Tables S11–S13). Our results showed that transcription regulatory factors were upregulated in the early stages of MPIV infection. The precise regulatory mechanism between the sponge effect and the expression of transcription regulatory factors remains unclear, but we showed the possible mechanism, and the upregulation of transcription regulatory factors may contribute to the subsequent upregulation of the expression of genes associated with an immune response against viral infections. We showed not only species- or tissue-specific interaction patterns of endogenous miRNAs with viral RNA but also a possible common mechanism conserved in human and mouse lungs for the regulation of endogenous gene expression during viral infection.

## DISCUSSION

Viral infectivity depends on multiple factors, such as receptors on the cell surface, subsequent membrane fusion, or environmental conditions. However, the mechanism by which the differential expression of endogenous miRNAs contributes to viral replication or gene expression remains unclear. In this study, we investigated the possible interaction between viral RNAs and endogenous miRNAs and the subsequent effects on the gene expression in human and mouse lungs. It has been reported that the miRNA profiles are adjusted over time in the middle and late stages of infection in response to the severity of the viral infection (Takahashi et al., 2021, 2018, 2020; Zhang et al., 2018). Our analysis showed that the original expression profiles of miRNAs before viral infection contributed to antiviral defense systems during the early stages of viral infection.

We analyzed “viral RNAs versus miRNAs” and visualized their possible interactions (Figs 1 and 2). Extensive interaction with endogenous miRNAs was observed in both MPIV and HPIV genomes and their complementary strands (Fig. 2); however, some genomic regions showed higher interaction counts than peripheral regions, whereas the others showed no interaction. Notably, the F gene of MPIV or HPIV showed species-specific differences, respectively (Fig. 3). Furthermore, we generated a list of miRNAs with multiple interaction sites on the viral RNA (Tables S3–S6). We visualized the possible interaction sites of miRNAs with multiple interaction sites (≥10) on the viral genome and its complementary strand (Fig. 4). We distinguished three types of miRNAs: common miRNAs in humans and mice, human-specific miRNAs, and mouse-specific miRNAs (Fig. 1). We analyzed how the interactions between viral RNAs and endogenous miRNAs influence endogenous gene expression in host cells (Fig. 5). Our results suggested the possibility that transcription regulatory factors can be upregulated by the interaction between viral RNAs and miRNAs (Fig. 6). The upregulation of transcription regulatory factors may contribute to the regulation of antiviral cytokines, such as IFN, and antiviral genes, such as ISGs, to establish an antiviral state comprising endogenous gene expression in virus-infected cells.

Protein- or cell-guided immunity has been developed in mammals; however, the function of RNA-guided immunity is controversial. siRNA-guided antiviral immunity is an essential defense mechanism against viral infections in plants and invertebrates. Viral siRNAs exhibit perfect complementary sequences against viral RNA because they are generated from viral dsRNA, whereas endogenous miRNAs exhibit partial complementary sequences against viral RNA. Both viral siRNAs and endogenous miRNAs are loaded onto RISC. However, viral siRNA mediates the cleavage of viral RNA on AGO2, whereas miRNA induces RNA degradation and/or translational repression (Bartel, 2004; Wilson and Doudna, 2013). It has been reported that miRNAs are involved in mediating intrinsic mechanisms of viral RNA clearance from cells (Takahashi et al., 2021, 2018, 2020; Takahashi and Ui-Tei, 2020; Cullen, 2013; Girardi et al., 2018). Viral infection induces a variety of immune responses in humans, from asymptomatic to severe symptoms, but the regulatory mechanism of viral RNA clearance from cells, which differs among individuals, is unknown. Expression profiles of endogenous miRNAs differ not only among tissues but also among individuals (Li et al., 2018; Liu et al., 2021; Sonehara et al., 2022). Understanding when, where, and how miRNA-guided antiviral immunity functions in collaboration with protein- and cell-guided immunity in individuals is a key question for future research. Further investigation is necessary to reveal the differences in age or gender and the dependence on genetic polymorphisms in individuals. It is also important to investigate the differences in expression profiles and their antiviral effects between tissues. Viruses have evolved to escape the efficient antiviral immunity of their hosts. The species-specific difference in the F gene of MPIV or HPIV could be possibly one of the escape mutations (Fig. 3). An approach that traces the evolution of viruses is intriguing.

In summary, we showed both species- and tissue-specific possible interaction between viral RNAs and endogenous miRNAs and common mechanism conserved in humans and mice by comparing endogenous miRNAs expressed in human or mouse lungs. Further experimental investigation is necessary to understand the complete mechanism of miRNA-guided immunity.

## MATERIALS AND METHODS

### Data of small RNA-seq and viral RNA sequence

The small RNA-seq data of samples acquired from human lungs or mouse lungs were obtained from a public database (Sequence read archive, SRA). The data were classified by age: human 15 donors aged 16–39 years and 23 mice aged 8–16 weeks (Table S2). The viral RNA sequences were obtained from NCBI: MPIV Cantell strain (GenBank: AB855653.1) and HPIV1/USA/38081A/2011 (GenBank: KF530212.1).

### Analysis of small RNA-seq data

For miRNA analysis, 18–25 nucleotide (nt)-long reads were extracted following the trimming of adaptor sequences using Cutadapt (version 1.18). The quality of the data was confirmed using FastQC (version 0.11.9). The 18–25 nt-long reads were mapped onto the human genome (GRCh38.p14) or mouse genome (GRCm38.p6) with miRNA annotation data obtained from miRBase (version 22) using STAR (version 2.7.4a). Read counts were calculated using featureCounts (version 2.0.3). Reads mapped to multiple locations simultaneously were counted as one read when all mapped locations were added. The read counts were counted as transcripts per million (TPM).

### Analysis of the interaction between viral RNAs and miRNAs

To extract the common pattern of specific generation in human or mouse lungs, the TPM values of each miRNA were averaged across the dataset. As the first filter of the analysis, miRNAs expressed in human or mouse lungs (TPM ≥100) were obtained from all miRNAs registered on miRBase (version 22). As the second filter, miRNAs that were assumed to be effective in terms of nucleotide preference at the 5′ end of mature miRNAs were obtained. As the third filter, the miRNAs that formed base pairs with viral RNAs via the seed region, which was present in positions 2–7 from the 5′ end of the miRNA, were obtained. Visualization of the data was performed using R (version 4.2.0). Target prediction of miRNA and gene ontology (GO) analysis were performed using TargetScan (Agarwal et al., 2015; McGeary et al., 2019) and DAVID (Huang et al., 2009; Sherman et al., 2022) online software tools, respectively.

### Cell culture

A549 cells, which are cultured cells derived from human lungs, were obtained from the JCRB cell bank (#JCRB0076) and were cultured in Dulbecco's Modified Eagle's medium (Wako) containing 10% fetal bovine serum (Gibco) and antibiotics (100 U/ml of penicillin and 100 µg/ml of streptomycin, Sigma-Aldrich) at 37°C in an atmosphere containing 5% CO_2_.

### Viral infection

MPIV cantell strain was incubated in a serum-free medium for 1 h at 37°C in an atmosphere containing 5% CO_2_. After adsorption, the medium was replaced, and the cells were cultured in a serum-containing medium at 37°C in an atmosphere containing 5% CO_2_.

### Gene expression profiling of MPIV-infected A549 cells

Cells were collected at 0, 1, 2, and 6 h following viral infection. Total RNA was extracted using a FastGene RNA Premium Kit (Nippon Genetics). The quality of total RNA was confirmed using a bioanalyzer. The sequence library was prepared using the TruSeq stranded mRNA library preparation kit. Sequencing was performed using NovaSeq6000 with 100 bp paired ends (Macrogen).

## Supplementary Material

10.1242/biolopen.060172_sup1 Supplementary information

Table S1. The number of protein-coding genes and miRNAs in each species

Table S2. The small RNA-seq data used in this study

Table S3. The list of human lung miRNAs with multiple interaction sites on the MPIV genome and its complementary strand

Table S4. The list of mouse lung miRNAs with multiple interaction sites on the MPIV genome and its complementary strand

Table S5. The list of human lung miRNAs with multiple interaction sites on the HPIV genome and its complementary strand

Table S6. The list of mouse lung miRNAs with multiple interaction sites on the HPIV genome and its complementary strand

Table S7. The GO analysis of genes that can be dysregulated by MPIV infection in human lungs

Table S8. The GO analysis of genes that can be dysregulated by MPIV infection in mouse lungs

Table S9. The GO analysis of genes that can be dysregulated by HPIV infection in human lungs

Table S10. The GO analysis of genes that can be dysregulated by HPIV infection in mouse lungs

Table S11. The GO analysis of 60 upregulated genes in MPIV-infected A549 cells (1 h, SeV/mock > 2).

Table S12. The GO analysis of 209 upregulated genes in MPIV-infected A549 cells (2 h, SeV/mock > 2).

Table S13. The GO analysis of 889 upregulated genes in MPIV-infected A549 cells (6 h, SeV/mock > 2).

## First person

This article has an associated First Person interview with Yoshiaki Kitsu, co-first author of the paper.
