# Supplementary material for: microRNA-guided immunity against respiratory virus infection in human and mouse lung cells
Source: Biol Open. 2024 Jun 17;13(6):bio060172. doi: 10.1242/bio.060172 (PMC11212637; doi:10.1242/bio.060172)
Supplement: Supplementary information [file biolopen-13-060172-s1.pdf]

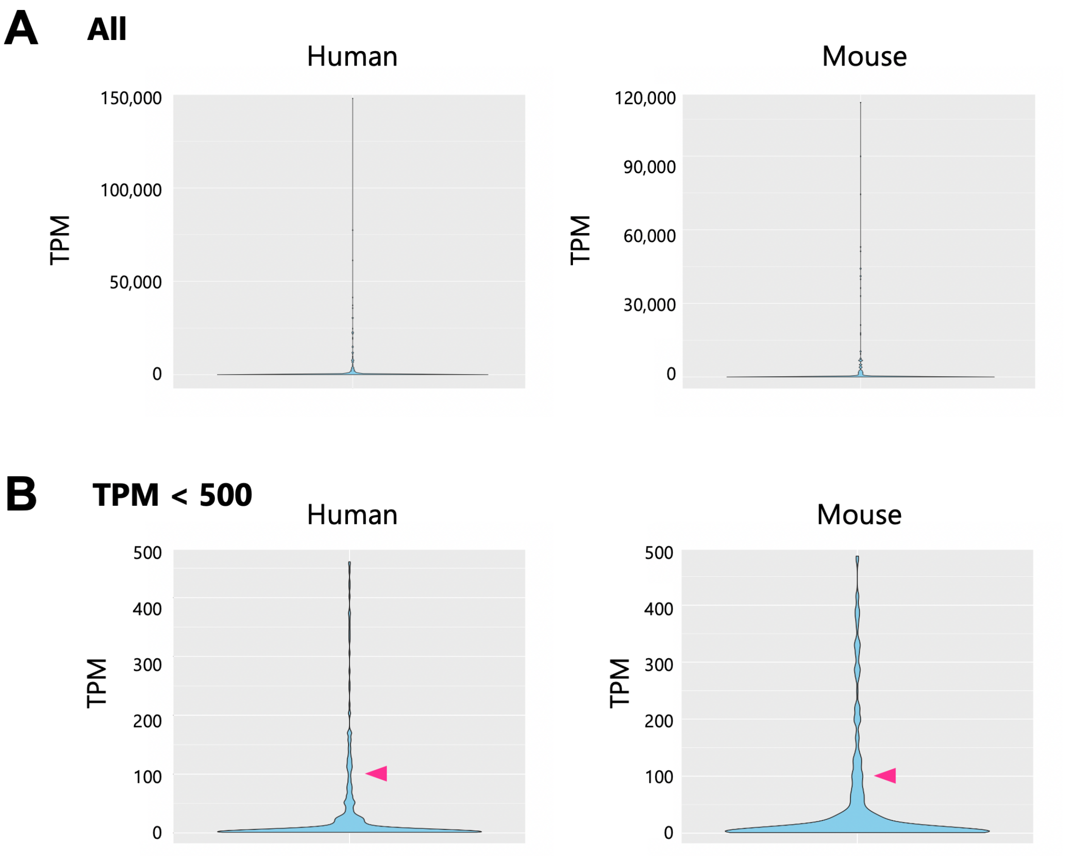

Supplementary Figure S1. Violin plots of the expression levels of miRNAs expressed in human or mouse lungs

(**A**) Violin plots of the expression levels of all miRNAs expressed in human or mouse lungs with TPM values ≥ 1. (**B**) Violin plots of the expression levels of miRNAs expressed in human or mouse lungs with TPM values ≥ 1, with an expanded range including TPM values below ≤ 500. Pink arrowheads indicate TPM values = 100, which is set as the threshold for effective miRNAs.

Biology Open • Supplementary information

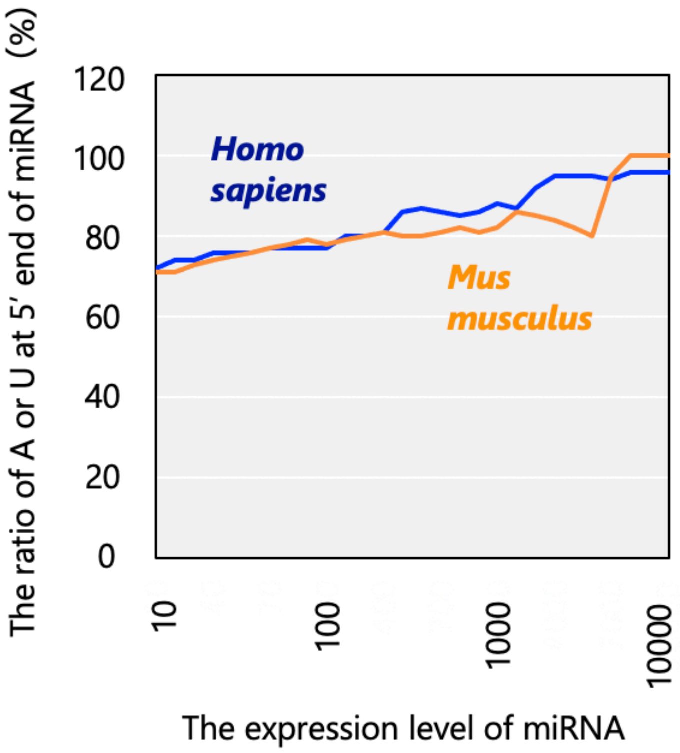

Supplementary Figure S2. Correlation between the expression and the ratio of 5' nucleotide (U or A).

A positive correlation between the expression and the ratio of the 5' nucleotide (U or A is) clearly observed.

Biology Open • Supplementary information

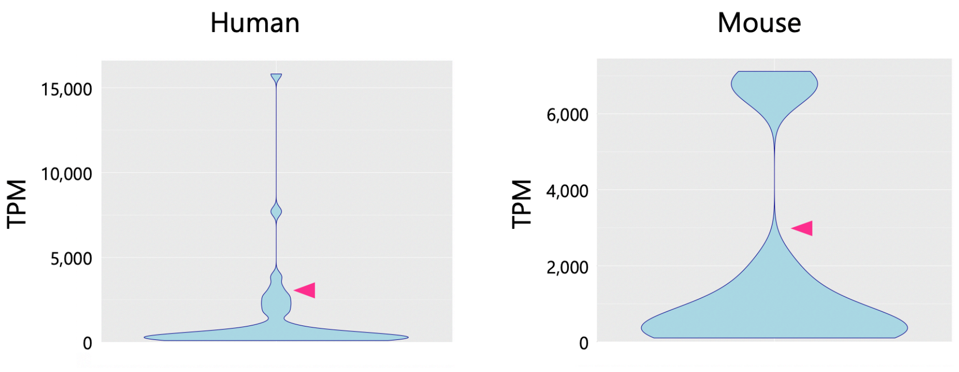

Supplementary Figure S3. Violin plots of the expression levels of miRNAs whose 5' end is C or G.

Violin plots of the expression levels of miRNAs whose 5' end is C or G expressed in human or mouse lungs with TPM values ≥ 1. Pink arrowheads indicate TPM values = 3000, which is set as the threshold for effective miRNAs whose 5' end is C or G.

Biology Open • Supplementary information

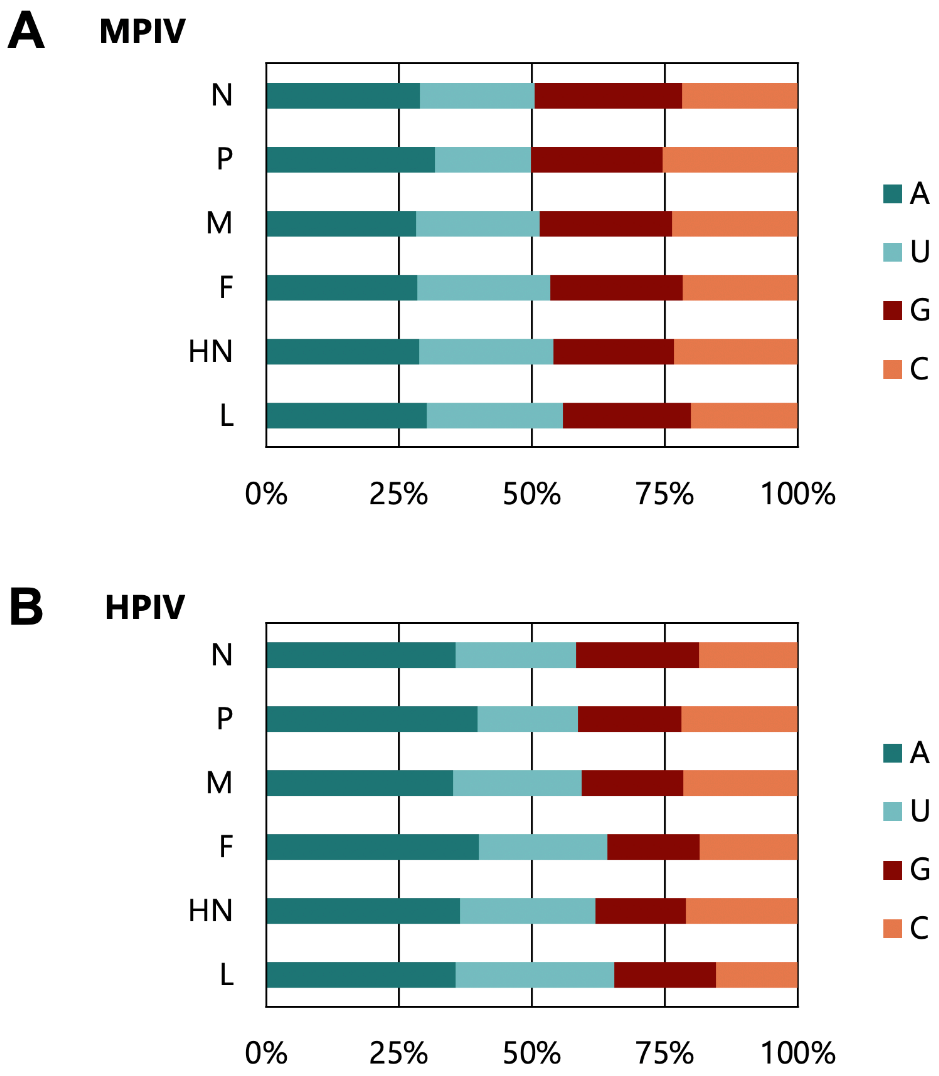

Supplementary Figure S4. The nucleotide contents of genes encoded in MPIV or HPIV. The nucleotide contents of genes encoded in MPIV or HPIV were analyzed, and the ratio of each nucleotide was shown in a different color.

Biology Open • Supplementary information

Table S1. The number of protein-coding genes and miRNAs in each species

Available for download at
https://journals.biologists.com/bio/article-lookup/doi/10.1242/bio.060172#supplementary-data

Table S2. The small RNA-seq data used in this study

Available for download at
https://journals.biologists.com/bio/article-lookup/doi/10.1242/bio.060172#supplementary-data

Table S3. The list of human lung miRNAs with multiple interaction sites on the MPIV genome and its complementary strand

Available for download at
https://journals.biologists.com/bio/article-lookup/doi/10.1242/bio.060172#supplementary-data

Table S4. The list of mouse lung miRNAs with multiple interaction sites on the MPIV genome and its complementary strand

Available for download at
https://journals.biologists.com/bio/article-lookup/doi/10.1242/bio.060172#supplementary-data

Table S5. The list of human lung miRNAs with multiple interaction sites on the HPIV genome and its complementary strand

Available for download at
https://journals.biologists.com/bio/article-lookup/doi/10.1242/bio.060172#supplementary-data

Table S6. The list of mouse lung miRNAs with multiple interaction sites on the HPIV genome and its complementary strand

Available for download at
https://journals.biologists.com/bio/article-lookup/doi/10.1242/bio.060172#supplementary-data

Table S7. The GO analysis of genes that can be dysregulated by MPIV infection in human lungs

Available for download at
https://journals.biologists.com/bio/article-lookup/doi/10.1242/bio.060172#supplementary-data

Table S8. The GO analysis of genes that can be dysregulated by MPIV infection in mouse lungs

Available for download at
https://journals.biologists.com/bio/article-lookup/doi/10.1242/bio.060172#supplementary-data

Table S9. The GO analysis of genes that can be dysregulated by HPIV infection in human lungs

Available for download at
https://journals.biologists.com/bio/article-lookup/doi/10.1242/bio.060172#supplementary-data

Table S10. The GO analysis of genes that can be dysregulated by HPIV infection in mouse lungs

Available for download at
https://journals.biologists.com/bio/article-lookup/doi/10.1242/bio.060172#supplementary-data

Table S11. The GO analysis of 60 upregulated genes in MPIV-infected A549 cells (1 h, SeV/mock > 2).

Available for download at
https://journals.biologists.com/bio/article-lookup/doi/10.1242/bio.060172#supplementary-data

Table S12. The GO analysis of 209 upregulated genes in MPIV-infected A549 cells (2 h, SeV/mock > 2).

Available for download at
https://journals.biologists.com/bio/article-lookup/doi/10.1242/bio.060172#supplementary-data

Table S13. The GO analysis of 889 upregulated genes in MPIV-infected A549 cells (6 h, SeV/mock > 2).

Available for download at
https://journals.biologists.com/bio/article-lookup/doi/10.1242/bio.060172#supplementary-data
